# Salt Stress Tolerance, Antioxidant Response, and the Effectiveness of Seed-Applied Stress Attenuators in Pumpkin Cultivars

**DOI:** 10.3390/plants15192920

**Published:** 2026-09-24

**Authors:** Daise Feitoza da Rocha, Kleane Targino Oliveira Pereira, Maria Valdiglezia de Mesquita Arruda, Salvador Barros Torres, Clarisse Pereira Benedito, Giovanna Dias de Sousa, Emanuele Lucas Moura, Iracema de Azevedo Monte Paiva, Jéssica Christie Dantas de Oliveira Costa, Pablo Ferreira da Silva, Roseane Rodrigues de Oliveira, Emerson de Medeiros Sousa, Angie Alejandra Rodriguez Cruz, José Eduardo Santos Barboza da Silva, Marciana Bizerra de Morais

**Affiliations:** 1 Department of Agricultural and Forestry Sciences, Federal Rural University of the Semi-Arid Region, Mossoró 59625-900, RN, Brazil; kleane_rn@hotmail.com (K.T.O.P.); valarruda2015@gmail.com (M.V.d.M.A.); sbtorres@ufersa.edu.br (S.B.T.); clarisse@ufersa.edu.br (C.P.B.); giovanna.sousa@alunos.ufersa.edu.br (G.D.d.S.); emanuelemoura8@gmail.com (E.L.M.); iracema.paiva@alunos.ufersa.edu.br (I.d.A.M.P.); chris_jessica2011@hotmail.com (J.C.D.d.O.C.); pablo.silva02405@alunos.ufersa.edu.br (P.F.d.S.); roseane.oliveira@alunos.ufersa.edu.br (R.R.d.O.); emerson.sousa@ifrn.edu.br (E.d.M.S.); alejandra.rdrgz17@gmail.com (A.A.R.C.); 2 Federal Institute of Education, Science and Technology of Bahia, Xique-Xique 47404-899, BA, Brazil; jose.eduardo@ifbaiano.edu.br; 3 Psychology Course, UniCatólica of Rio Grande do Norte, Mossoró 59625-900, RN, Brazil; marciana.bio@gmail.com

**Keywords:** *Cucurbita* spp., salt stress, seed pre-treatment, oxidative stress, stress mitigators

## Abstract

Salinity compromises seed germination and early seedling growth, highlighting the need to identify tolerant cultivars and effective management strategies. This study aimed to evaluate tolerance to salt stress, antioxidant activity, and the effectiveness of seed-applied stress attenuators in pumpkin cultivars. The research was conducted in two stages. In the first stage, six cultivars (Tetsukabuto, Soberana, Kin, Bahiana Tropical, Sergipana, and Adele) were subjected to three levels of osmotic potential induced by NaCl (0.0, −0.2, and −0.4 MPa). Germination, growth, and biochemical variables were evaluated. In the second stage, two contrasting cultivars were subjected to six treatments: control (0.0 MPa), salt stress (−0.4 MPa), hydropriming, gibberellic acid, ascorbic acid, and salicylic acid, all under salt stress (−0.4 MPa). In addition to germination and growth variables, oxidative damage (H_2_O_2_ and lipid peroxidation) and antioxidant activity (SOD, CAT, and APX) were evaluated. Salt stress significantly reduced germination, growth, and biomass accumulation. Cultivar Adele showed greater tolerance, whereas Tetsukabuto was more sensitive. Hydropriming, gibberellic acid, ascorbic acid, and salicylic acid mitigated the effects of salinity, with hydropriming showing the greatest reduction in lipid peroxidation. It is concluded that Adele, combined with gibberellic acid, and Tetsukabuto, combined with hydropriming and gibberellic acid, showed better performance under salinity stress, highlighting the potential of physiological and biochemical traits for identifying contrasting responses to salinity.

## 1. Introduction

Pumpkin is a functional fruit vegetable that can be consumed as a medicinal, nutritious, and multifunctional food due to its high contents of essential minerals and phytochemicals, such as β-carotene, total flavonoids, and total phenolics, which contribute to delaying aging and strengthening the immune system [1]. It is also an economically important crop, moderately sensitive to salinity, with growth directly influenced by saline soil conditions [2].

Soil salinization has become a serious global agricultural problem, especially in arid and semi-arid regions, compromising sustainable development goals related to food security, agriculture, resource conservation, and nutrition [3]. In these regions, salinization associated with water scarcity has contributed to reduced agricultural productivity and the productive capacity of irrigated systems.

Salinity impairs plant growth by lowering soil osmotic potential, limiting root water uptake and cellular hydration, which leads to physiological imbalances, reduced photosynthesis, and diminished growth; it also affects germination and early development [4,5]. Salt stress generates reactive oxygen species (ROS) that damage macromolecules such as lipids and proteins and, together with high concentrations of Na^+^ and Cl^−^ ions, compromise vital metabolic functions, including enzymatic activities and photosynthetic efficiency [6,7,8].

Integrating the conceptual framework of salt stress-response mechanisms with strategies to increase tolerance in cultivated plants is essential [9]. This tolerance is mediated by several biochemical pathways that favor water uptake and maintain cellular ionic homeostasis, allowing plants to adapt to saline environments [10]. Proline, for example, supports osmotic adjustment, whereas soluble sugars act as osmoprotectants essential for defense against abiotic stress [11]. In this context, seed priming emerges as a promising alternative, using the plant’s stress memory and activating its immune system to stimulate germination and promote growth under abiotic stresses, without the need for genetic engineering [12]. Among the techniques already investigated, seed hydropriming is one of the most studied for vegetable species [13].

Complementarily, the exogenous application of phytohormones has also proven effective in coping with environmental stresses, including salinity. In this context, the use of attenuators such as salicylic acid (SA) and ascorbic acid (AsA) has been associated with increased plant stress tolerance [14,15]. In cucurbits, recent studies demonstrate that seed treatment with salicylic acid and oxidative compounds can increase the activity of antioxidant enzymes and reduce oxidative damage, while gibberellic acid contributes to osmotic adjustment through the accumulation of compatible solutes [5]. Furthermore, gibberellic acid (GA_3_) can improve photosynthesis, growth, and water adjustment in plants under salinity conditions [16].

The use of attenuators has helped mitigate salt stress by increasing ROS scavenging capacity and the activity of antioxidant enzymes [17]. The antioxidant enzyme system plays a crucial role in plant defense, being responsible for maintaining reactive oxygen species homeostasis in cells and ensuring normal plant growth and development after salt stress [18].

Thus, it is believed that pre-treating pumpkin seeds with seed priming agents such as hydropriming, gibberellic acid, salicylic acid, and ascorbic acid may enhance plant adaptive capacity by promoting greater antioxidant activity and mitigating salinity-induced damage. Therefore, this study aimed to evaluate salt stress tolerance, antioxidant activity, and the effects of seed pre-treatment in different pumpkin cultivars, aiming to overcome the limitations imposed by salinity conditions.

## 2. Results

### 2.1. Stage I

According to the analysis of variance, there is a significant interaction (*p* < 0.01) between factors (cultivars × salt stress levels) for first germination count, germination, germination speed index, shoot length, root length, and shoot dry mass. Root dry mass, in contrast, showed isolated effects of both factors (Table 1).

Reductions in first germination count (FGC), germination (G), and germination speed index (GSI) varied according to cultivar and salinity level (Table 2). Among the cultivars, Adele obtained the best results for FGC, G, and GSI at both saline levels; however, among these levels, the highest means for FGC, G, and GSI, 94%, 98%, and 12.11, respectively, were observed at the −0.2 MPa level (Table 2). Thus, cultivar Adele stood out as a promising candidate for salt stress tolerance. Germination (G) of cv. Tetsukabuto was reduced by 29.1% and 11.6% at the −0.2 and −0.4 MPa levels, respectively, compared with the control (Table 2).

Salt stress reduced shoot length (SL), root length (RL), shoot dry mass (SDM), and root dry mass (RDM). For SL and RL, at the −0.2 MPa potential, the cultivars Tetsukabuto and Soberana obtained lower results; however, they did not differ from Sergipana in root length (Table 3). The −0.4 MPa salinity level reduced SL and RL in pumpkin seedlings, with no statistical differences among cultivars (Table 3).

The SDM results indicated that the lowest values were obtained for cvs. Tetsukabuto, Bahiana Tropical, and Sergipana at the −0.4 MPa level. At the −0.2 MPa level, cv. Adele stood out with higher SDM, while at the −0.4 MPa level, it did not differ from cvs. Soberana and Kin (Table 3).

For the isolated effect of root dry mass (RDM), the highest value was observed in the absence of salt stress, representing the most favorable condition for root growth (Figure 1). As the osmotic potential decreased to −0.2 MPa, root dry mass was significantly reduced by 20%. At the more severe osmotic potential of −0.4 MPa, root dry mass was approximately 36% lower than in the control.

Differences in root dry mass among the six pumpkin cultivars were independent of salt stress. Cv. C3 (Kin) obtained the best performance, with higher root dry mass, followed by C2 (Soberana) and C4 (Adele), which also resulted in superior dry masses, indicating intermediate performance. Cv. C5 (Bahiana Tropical) resulted in lower root dry mass, being statistically inferior to the others. Cvs. C1 (Tetsukabuto) and C6 (Sergipana) did not differ from each other (Figure 1).

For the selection of the salinity level to be used in the second experimental stage, it was considered that the −0.2 and −0.4 MPa levels had different means, so it was preferable to use the −0.4 MPa level in phase two. This salinity reduced both root and shoot growth in the cultivars (Table 3).

Representative seedlings are shown in Figure 2. Increasing salinity reduced seedling growth in all cultivars, particularly at −0.4 MPa. Differences in shoot and root development among cultivars were visually evident, with cv. Adele showing greater growth maintenance under salt stress, whereas cv. Tetsukabuto showed a more pronounced reduction in seedling development. The other cultivars displayed intermediate responses.

Analysis of variance for the quantification of total soluble sugars, total free amino acids, free proline, and free citrulline indicated an interaction between cultivars and osmotic potentials for salt stress (Table 4).

Increasing salinity levels promoted osmotic homeostasis in pumpkin cultivars. TSS concentrations increased at the −0.2 MPa level, except for cv. Adele, which remained stable at all salinity levels (Table 5). Increases of 76.5, 48.0, 46.6, 6.0, 176.9, and 131.2% were observed for cultivars Tetsukabuto, Soberana, Kin, Adele, Bahiana Tropical, and Sergipana, respectively. Among cultivars, the highest TSS content was determined in Tetsukabuto at the −0.2 MPa level (Table 5).

Quantification of TFAA at the no-salinity level for cv. Tetsukabuto resulted in a higher amount of amino acids, followed by cv. Kin (Table 5). With increasing salinity to −0.2 MPa, the pumpkin cultivars increased TFAA synthesis, except for Tetsukabuto and Sergipana, which reduced TFAA. At the highest salinity (−0.4 MPa), cv. Bahiana Tropical produced more TFAA, with a gain of 184.6% compared to the control and 99.1% compared to cv. Tetsukabuto at the same salinity level (Table 5).

For proline, cv. Tetsukabuto stood out compared to the other cultivars. However, at the moderate salinity level (−0.2 MPa), proline content decreased and then increased at −0.4 MPa by 46.9% (Table 6). Citrulline, another amino acid associated with stress response, was produced in greater quantity in cv. Adele, mainly at −0.4 MPa potential, with a gain of 89.5%, not differing from cv. Bahiana Tropical. Across salinity levels, cvs. Tetsukabuto and Sergipana showed no variation in citrulline, indicating a possible different stress-response strategy (Table 6).

In the dendrogram, Euclidean distance was used to measure 30% dissimilarity among combinations of pumpkin cultivars (C) and osmotic potential levels (N) under salt stress (Figure 3). The analysis allowed the formation of groups that reflect the similarity or dissimilarity among the tested combinations. Group I clustered the cultivars without salt stress (0.0 MPa), demonstrating that these combinations are similar in terms of germination and performance of biochemical and growth variables (Figure 3).

The second group included cultivar Adele, the most salt-tolerant cultivar, as it showed the best performance at −0.4 MPa. Group III contained salt-sensitive cultivars; Tetsukabuto and Soberana were farthest from the others, indicating greater sensitivity to salt stress (Figure 3). Based on these results, the saline restriction level of −0.4 MPa was selected for the second phase of this study, with cultivars Adele and Tetsukabuto identified as the most tolerant and sensitive to salt stress, respectively (Figure 3). The dendrogram reinforces this distinction by showing a clear separation between the most tolerant and most sensitive cultivars to salt stress, with some of them clustering near the control condition, indicating good performance even under salinity.

### 2.2. Stage II

Analysis of variance revealed a significant cultivar × attenuator interaction (*p* < 0.05) for germination, shoot length, and shoot dry mass and a highly significant interaction (*p* < 0.01) for germination speed index and root dry mass (Table 7). First germination count showed an isolated effect for cultivar (*p* < 0.01). Root length was influenced only by treatments with stress attenuators (*p* < 0.01) (Table 7).

Cv. Adele had a higher first-count germination, 7.09% greater than cv. Tetsukabuto; however, both exceeded 80% germination on the fourth day after sowing (Figure 4A). In the control, the cultivars showed similar germination, averaging 88%, but under −0.4 MPa, this variable decreased by 13.08% only in cv. Tetsukabuto relative to the control. After seeds were treated with ascorbic acid (AsA) and salicylic acid (SA), germination percentages were similar to the control, even under salt stress (Figure 4B). For cv. Adele, all treatments maintained germination above 85%. The difference between cultivars was identified only in the saline treatment without attenuators (SS) and when seeds were pre-treated with water (H_2_O) and gibberellic acid (GA_3_). Cv. Adele stood out, obtaining superior results to cv. Tetsukabuto (Figure 4B).

For the germination speed index (GSI), in all treatments, cv. Adele had higher values than Tetsukabuto, indicating greater tolerance to the applied salinity (Figure 4C). In cv. Tetsukabuto, GSI decreased by 32.08% under salt stress and remained reduced even after pre-treatment with stress attenuators, averaging 9.13 (Figure 4C).

The highest salt stress level (−0.4 MPa NaCl) significantly reduced the shoot length of pumpkin cultivars compared with the control. The control averaged 5.06 cm, whereas salt stress caused a 62.45% reduction (Figure 5A). For cv. Tetsukabuto, treatments with attenuators (H_2_O, GA_3_, AsA, and SA) under salt stress promoted partial recovery, increasing shoot length by 27.07% when compared to stress alone, with averages above 2.40 cm (Figure 5A). For Adele, the pre-germinative treatments promoted partial shoot growth but did not differ from salt stress.

For root growth, regardless of cultivar, the control produced longer roots, averaging 12.26 cm, whereas salt stress reduced length by 14.68% to 10.46 cm seedling^−1^ (Figure 5B). At the concentrations used, the stress attenuators (H_2_O, GA_3_, AsA, and SA) did not fully restore root length lost to salinity, with an average of 10.1 cm seedling^−1^. Therefore, although not differing from stress alone, root length remained 17.62% lower than the control (Figure 5B).

For dry mass, responses differed from those for seedling length (Figure 6A). Shoot dry mass (SDM) was similar between cultivars in the control. Salt stress reduced growth, with losses of 40.9% for cv. Tetsukabuto and 37% for cv. Adele. For SDM, GA_3_ yielded the best result for cv. Tetsukabuto, with a 56.2% gain relative to salt stress. For cv. Adele, no attenuator mitigated the salinity effect on SDM (Figure 6A). Between cultivars, GA_3_ and SA produced distinct SDM responses, with cv. Tetsukabuto outperforming cv. Adele by 27.7% and 25%, respectively (Figure 6A).

Root dry mass (RDM) in the control was similar among the pumpkin cultivars. Under salt stress, cultivars responded differently (Figure 6B). Cv. Tetsukabuto showed a 26.72% increase in RDM under salt stress compared to the control. In this cultivar, GA_3_ resulted in a 32.7% increase compared to the absence of salts (Figure 6B). In cv. Adele, salt stress reduced RDM by approximately 20.84% compared to the control. Among the attenuator treatments, for this cultivar, there was no difference in RDM compared to the control, with an average of 9.7 g seedling^−1^, except when using SA, which further reduced RDM, reaching 8.5 g seedling^−1^. Among cultivars, only GA_3_ and SA promoted distinct responses, with emphasis on cv. Tetsukabuto (Figure 6B).

Analysis of variance revealed significant interaction effects (*p* < 0.01) between cultivar and attenuator treatment factors for total soluble sugars, total free amino acids, free proline, and free citrulline (Table 8).

For total soluble sugars (TSS), the cultivars showed values statistically similar to the control (Figure 7A). Under salinity, cv. Tetsukabuto exhibited a significant 46.3% increase in TSS relative to the control. In this cultivar, AsA was the most effective attenuator, promoting a 126.5% increase in TSS compared to the absence of salts (Figure 7A). In cv. Adele, there was no difference in TSS quantification among treatments, with an average of 27.3 mg TSS g^−1^ FM (Figure 7A).

For free amino acids (Figure 7B), salt stress also caused a significant increase compared to the control of 29.43% and 53.73% for cultivars Tetsukabuto and Adele, respectively. The hydropriming treatment (H_2_O) stood out for this variable, with an increase of 58.52% and 71.12% compared to stress alone for Tetsukabuto and Adele, respectively. This effect was followed by AsA and SA, which also produced smaller increases in cv. Tetsukabuto (Figure 7B).

Proline levels were also higher under salt stress compared to the control, with increases of 66.66% and 200% for cvs. Tetsukabuto and Adele, respectively (Figure 7C). Among the attenuators, hydropriming maintained the proline level similar to that obtained under salt stress for cv. Tetsukabuto and promoted a 200% increase in cv. Adele (Figure 7C). With GA_3_, seedlings of both cultivars showed similar values at 0.0017 µmol PRO g^−1^ FM (Figure 7C).

The two cultivars differed in citrulline levels even in the control, with cv. Adele presenting higher values across all treatments (Figure 7D). Salt stress markedly increased citrulline relative to the control by 200% and 64.70% in cvs. Tetsukabuto and Adele, respectively. Salicylic acid (SA) was the treatment that most favored citrulline accumulation, with an increase of 17.85% compared to stress without attenuation for Adele, followed by ascorbic acid (AsA) (Figure 7D).

Analysis of variance revealed significant cultivar × treatment interaction effects (*p* ≤ 0.01) for all variables, indicating that the antioxidant response to salt stress in *Cucurbita* spp. is influenced by the attenuators (Table 9).

Hydrogen peroxide (H_2_O_2_) production was high in cv. Tetsukabuto even without salts, and it was reduced by the other treatments. Under stress, H_2_O_2_ decreased by 69.5% compared to the control (Figure 8A). Among the attenuator treatments, gibberellic acid (GA_3_) and ascorbic acid (AsA) reduced H_2_O_2_ levels, reducing them by approximately 76.85% in cv. Tetsukabuto (Figure 8A). In cv. Adele, salt stress increased H_2_O_2_ production by 105.26%. Among the attenuator treatments, salicylic acid (SA) promoted a reduction in H_2_O_2_ levels by 51.92% compared with stress without attenuators, not differing from the control (Figure 8A).

Malondialdehyde (MDA) levels were similar between cultivars in the control treatment (Figure 8B). Salt stress caused a significant 28.07% reduction relative to the control in cv. Adele, but MDA remained elevated in cv. Tetsukabuto (Figure 8B). Among the attenuators, hydropriming (H_2_O) most effectively reduced MDA by approximately 96.96% and 23.55% compared with stress alone in Tetsukabuto and Adele, respectively. Gibberellic acid (GA_3_), ascorbic acid (AsA), and salicylic acid (SA) produced smaller yet significant reductions in Tetsukabuto of 36.2%, 65.3%, and 81%, respectively (Figure 8B).

Catalase (CAT) activity declined under salt stress in both cultivars relative to the control by approximately 40.86% and 77.59% in Tetsukabuto and Adele, respectively. Among the attenuator treatments, hydropriming (H_2_O) for cv. Tetsukabuto and salicylic acid for cv. Adele promoted increases of 45.97% and 209.6%, respectively (Figure 9A). Among cultivars, only salt stress without an attenuator and with hydropriming maintained CAT levels statistically similar (Figure 9A).

For ascorbate peroxidase (APX), activity dropped sharply under stress compared with the control by approximately 33% and 82% in Tetsukabuto and Adele, respectively (Figure 9B). Among the stress attenuators, hydropriming (H_2_O) was able to recover APX activity for cv. Tetsukabuto, increasing it by 29.6% compared to stress without treatment, similar to the control. In cv. Adele, only ascorbic acid (AsA) was ineffective in maintaining APX activity, resulting in a 48% decrease compared with salt stress (Figure 9B).

For superoxide dismutase (SOD), the control treatment showed similar activities in both cultivars. In contrast, salt stress increased SOD activity relative to the control by 344% and 59% in cvs. Tetsukabuto and Adele, respectively (Figure 9C). All saline treatments with attenuators reduced SOD activity compared with salt stress. However, pre-treatments with gibberellic acid (GA_3_) for cv. Tetsukabuto and ascorbic acid (AsA) for cv. Adele promoted the lowest SOD activities, with a reduction of 93% and 91% compared to salt stress, respectively (Figure 9C).

Protein synthesis varied significantly between cultivars. Cv. Adele obtained a higher protein concentration in the control (83 mg g^−1^ FM protein), while cv. Tetsukabuto showed a lower value in this treatment (57 mg g^−1^ FM protein). Salt stress induced a significant increase in proteins in both cultivars, being 132% for cv. Tetsukabuto and 30% for cv. Adele, when compared to their respective controls (Figure 9D). In Tetsukabuto, all pre-germinative treatments yielded lower protein content than salt stress. Relative to the control, the highest values were observed with H_2_O (112 mg g^−1^ FM protein) and GA_3_ (106 mg g^−1^ FM protein), whereas AsA resulted in the lowest protein content (41 mg g^−1^ FM protein). In Adele, H_2_O produced the highest protein accumulation (146 mg g^−1^ FM protein), significantly exceeding the other treatments (Figure 9D).

Representative images of the seedlings visually support the quantitative results obtained for growth-related traits (Figure 10). Salt stress reduced shoot and root development in both cultivars, whereas pre-germinative treatments partially mitigated these effects, particularly in cv. Adele, which maintained a more vigorous seedling phenotype than cv. Tetsukabuto under saline conditions.

## 3. Discussion

### 3.1. Stage I

The cultivars differed in performance under saline stress. Cv. Adele showed higher germination, germination speed index, and proline accumulation even at the lowest osmotic potentials (−0.2 and −0.4 MPa), suggesting adaptive mechanisms involving osmotic adjustment and accumulation of osmoprotective compounds.

Plant adaptation to salinity involves multiple mechanisms, including root morphological changes, such as increased root density, that enhance water and nutrient uptake [19]. Physiological adjustments, notably osmotic adjustment mediated by increased levels of regulatory substances (proline, sugars, and proteins) [20,21], may have favored the germination process and initial growth of cv. Adele seedlings. This cultivar also exhibited higher citrulline levels at the highest salinity, suggesting a possible role of this compound in processes related to osmotic protection and mitigation of oxidative stress, contributing to the maintenance of cellular integrity under salinity [22].

Conversely, cv. Tetsukabuto was more sensitive to salt stress, with significant reductions in germination, first germination count, germination speed index, shoot and root length, and biomass. These results underscore this cultivar’s greater vulnerability under salinity, despite increased soluble sugar accumulation under moderate stress, which suggests an insufficient osmotic adjustment to sustain seedling growth. Similar findings were described by Taratima, Kunpratum and Maneerattanarungroj [23], who observed that salinity significantly reduced leaf growth, stem diameter, and root number in Laikaotok pumpkin.

The remaining cultivars (Soberana, Kin, Bahiana Tropical, and Sergipana) displayed intermediate or inconsistent responses across variables, precluding a clear classification for salt tolerance. For example, cv. Kin maintained high germination under stress but exhibited reduced growth and biomass; Soberana performed well for germination under moderate conditions but was highly sensitive in growth traits; and Bahiana Tropical and Sergipana showed pronounced biochemical responses (e.g., increased total free amino acids) yet poorer physiological performance, indicating a decoupling between biochemical and morphophysiological responses.

These results indicate that, although these cultivars exhibit partial stress-response mechanisms, they were insufficient to ensure consistent performance under salinity, limiting a clear classification as tolerant or sensitive. Although salt-tolerant genotypes generally show increased soluble sugar concentrations under saline conditions whereas sensitive genotypes often display lower concentrations, with reductions between 0.3% and 0.5% when exposed to NaCl [24], this pattern was not uniformly observed among the cultivars evaluated in this study.

The variability in responses, especially between physiological and biochemical parameters, reinforced by cluster analysis in the dendrogram, shows that sugar accumulation alone does not constitute an absolute indicator of tolerance, as some cultivars increased these compounds without maintaining germination and growth. This dissociation reinforces the complexity of salt stress responses and the need for an integrated approach to characterize tolerance. Accordingly, the distinction between tolerant and sensitive cultivars was based on the overall response profile, with cultivars Adele and Tetsukabuto selected as representatives of higher tolerance and sensitivity, respectively, for the next stage of this study, allowing a more in-depth investigation of the effects of salt stress attenuators on genotypes with different tolerance levels.

### 3.2. Stage II

Salt stress (−0.4 MPa NaCl) adversely affected biometric, biochemical, and enzymatic variables of pumpkin cultivars, whereas attenuator treatments promoted partial recovery, indicating potential to mitigate salt stress effects.

For germination variables, cv. Adele outperformed Tetsukabuto, suggesting a greater initial capacity to cope with salt stress. By contrast, the reduction in germination of cv. Tetsukabuto indicates impairment of essential physiological processes, possibly related to limited water uptake and metabolic disorganization. Adequate seed hydration is essential to initiate germination, and its restriction under salinity compromises reserve mobilization, protein synthesis, and cell division and is associated with oxidative stress, resulting in delayed emergence, reduced germination, seedling mortality, and impaired root development [8,25,26]. Similar results were reported by Tarchoun et al. [27] and Dantas et al. [28] in pumpkin varieties. In watermelon cultivars, salinity of −0.4 MPa reduced germination and germination speed index, with differences among cultivars regarding stability under stress [5].

The pre-germinative treatments H_2_O, GA_3_, AsA, and SA helped maintain germination and GSI, especially under salt stress, corroborating reports of positive effects of these compounds on initial seedling performance. According to Guirra et al. [29], increases in GSI in pumpkin seeds treated with plant growth regulators such as GA_3_ and SA are independent of the type of wastewater used. In cucurbits, the use of stress attenuators similar to those used in this research promotes positive effects on the germination process, as described by Leite et al. [5] in watermelon and by Souza Neta et al. [30] in cucumber.

Gibberellic acid and salicylic acid regulate growth and metabolic activation, promoting germination and cell elongation [29,31]. This effect was more evident in cv. Tetsukabuto, reflected in shoot length, whereas in Adele, variation under salt stress among attenuators was smaller, suggesting intrinsic tolerance.

Salt stress compromised the development of cultivars Tetsukabuto and Adele, reducing shoot and root length, likely due to stress-induced metabolic changes. Osmotic imbalance triggers biochemical responses characterized by the regulation of osmolyte biosynthesis, such as soluble sugars and proline, which contribute to maintaining plant water status and protecting cellular structures [20,32,33]. In addition, Na^+^ and Cl^−^ accumulation can contribute to cytotoxicity and nutritional imbalance, reducing plant growth [34]. Similar results were found by Dantas et al. [28] in pumpkin cultivars Coroa and Caserta and by Leite et al. [5] in watermelon cultivars.

Stress attenuators promoted partial growth recovery, mainly in cv. Tetsukabuto, possibly due to a priming effect, whereby pre-treatment activates defense mechanisms and induces stress memory, increasing resistance to subsequent stress events [33,35,36]. In cv. Adele, this effect was less expressive, reinforcing the lower dependence on external stimuli for this cultivar.

The reduction in shoot dry mass confirms the negative impact of salinity on plant growth, as also observed in zucchini by Canjá et al. [37]. Dry biomass is among the parameters most sensitive to salinity, decreasing markedly with increasing salinity and compromising mass accumulation while potentially causing cellular damage [38]. Gibberellic acid (GA_3_) stood out in recovering shoot dry mass of cv. Tetsukabuto, suggesting a potential effect in attenuating the impacts of salt stress. Silva et al. [34] found that, in Goldex melon seedlings, salicylic acid promoted superior results in shoot biomass production. Similarly, in watermelon cultivars, salicylic acid contributed to the recovery of shoot dry mass under water deficit, restoring values close to the control, although with variations among cultivars [39].

For root dry mass, the cultivars showed contrasting responses: a reduction in cv. Adele and an increase in Tetsukabuto under salt stress, indicating distinct adaptation strategies. Increased root biomass may represent a strategy for greater water exploration in the substrate, whereas the reduction in root biomass observed in cv. Adele may be associated with osmotic limitation and ionic toxicity [40,41].

Hydropriming (H_2_O), gibberellic acid (GA_3_), and ascorbic acid (AsA) stimulated the root development in cv. Adele, but values did not differ from those under salt stress. By contrast, in cv. Tetsukabuto, all treatments except AsA increased root biomass relative to the control (without stress). Preserving root development supports water uptake and mitigates stress effects. Salicylic acid (SA) can act by mitigating stress effects and partially recovering root growth inhibition [42]. However, this characteristic was not observed for cultivar Adele.

Patterns of osmolyte accumulation differed between cultivars. In Tetsukabuto, AsA pre-treatment increased soluble sugars, and hydropriming increased proline; in Adele, amino acids and citrulline were higher. These results indicate cultivar-specific metabolic responses to salt stress, consistent with distinct osmotic adjustment strategies. In agreement, previous studies reported sugar increases under salt stress and regulation by salicylic acid. Dantas et al. [28] observed accumulation of reducing sugars resulting from the degradation of total soluble sugars in pumpkin seed cotyledons during germination, particularly under salt stress. Sugar levels also rose in pumpkin (*Cucurbita pepo* L.) treated with SA [43] and in sorghum under salinity [44]. In *Cucurbita moschata* seedlings (cv. Sergipana) subjected to fish farming wastewater with EC of 5.97 dS/m, gibberellic and salicylic acids increased total sugars [29], and in watermelon cultivars, hydropriming and gibberellic acid increased soluble sugars under water stress [39], partially corroborating the present study.

Hydropriming (H_2_O) most strongly promoted amino acid accumulation in both cultivars and also lowered MDA. Consistent with this, Silva et al. [45] reported greater total amino acid accumulation in Grand Prix melon seedlings irrigated with saline water under gibberellic acid. Similarly, pre-treatment of basil seeds with regulators such as GA_3_, SA, or AsA also promoted an increase in amino acids, associated with the recovery of physiological parameters and osmotic adjustment [46], corroborating our results.

Proline, a key osmotic adjuster, increased in both cultivars under salt stress, with a stronger response in cv. Tetsukabuto, consistent with Tarchoun et al. [27], for pumpkin landraces. Proline contributes to structural stabilization, maintenance of membrane integrity, and reactive oxygen species detoxification [47,48]. Across pre-germinative treatments, cultivar differences in proline occurred only under salt stress and hydropriming (H_2_O); under GA_3_, AsA, or SA, levels were similar. Although SA did not stand out here, it has been associated with proline production, improved water uptake, and activation of antioxidant enzymes [49,50]. Overall, the use of attenuators as pre-germinative treatments can help mitigate the effects of salt stress, as observed in basil [46], favoring seedling establishment.

Citrulline increased significantly in both cultivars under salt stress, suggesting involvement in metabolic adjustment, particularly in cv. Adele. Although the results obtained do not allow for a direct determination of this metabolite’s role in salinity tolerance, previous studies have also reported its accumulation under stress conditions. Malambane et al. [51] observed a marked increase in citrulline in watermelon leaves subjected to adverse conditions and discussed its potential association with osmotic adjustment and the scavenging of reactive oxygen species. Similarly, Sewelo et al. [52] found that higher citrulline concentrations were associated with watermelon genotypes that performed better under water deficit. In this context, the greater accumulation of this amino acid in the Adele cultivar suggests a physiological response to salt stress, although further studies are needed to clarify its specific contribution to tolerance mechanisms.

Salicylic acid (SA) was the most effective treatment for the Adele cultivar, followed by ascorbic acid (AsA), promoting better performance under saline conditions. These results corroborate studies linking these compounds to the plant response to abiotic stress, noting their involvement in processes such as cell signaling, physiological regulation, and antioxidant protection [53].

Hydrogen peroxide (H_2_O_2_) levels were higher in the control treatment of the Tetsukabuto cultivar compared to treatments subjected to salt stress. Although H_2_O_2_ is frequently used as an indicator of oxidative stress, its interpretation requires caution, as this compound also acts as a signaling molecule in various physiological plant responses to stress. Thus, the differences observed between treatments may reflect changes in the dynamics of reactive oxygen species production and scavenging, as well as in cell signaling processes associated with adaptation to salt stress.

In the Adele cultivar, the highest H_2_O_2_ levels were reported in treatments involving hydropriming (H_2_O), gibberellic acid (GA_3_), and ascorbic acid (AsA). Conversely, the Tetsukabuto cultivar exhibited lower concentrations of this metabolite under salinity, alongside an increase in root dry mass and soluble sugar content. Although this association suggests distinct physiological responses between the cultivars, the results do not establish a direct causal relationship between H_2_O_2_ levels and salt stress tolerance. Previous studies report that maintaining reactive oxygen species homeostasis, rather than necessarily achieving their complete reduction, is a component of plant adaptation to salinity [18,32,54].

Among the mitigating agents evaluated, treatments with gibberellic acid (GA_3_) and ascorbic acid (AsA) were associated with the lowest H_2_O_2_ concentrations in the Tetsukabuto cultivar. Given that ascorbic acid directly participates in scavenging reactive oxygen species and that plant regulators can modulate antioxidant responses, this result suggests these substances may influence the regulation of oxidative metabolism during plant establishment under saline conditions. However, confirming these similar mechanisms requires an analysis of the enzymatic antioxidant system.

Treatment with salicylic acid (SA) reduced H_2_O_2_ production [53]. Similarly, previous studies report that gibberellic acid can decrease lipid peroxidation and electrolyte leakage and modulate H_2_O_2_-metabolizing enzymes, potentially contributing to membrane stability [55,56]. Although often associated with oxidative damage, H_2_O_2_ also functions as a signaling molecule, and its regulation is essential to balance the deleterious effects and adaptive mechanisms under salt stress [57]. These results reinforce the role of ascorbic acid and gibberellic acid in regulating oxidative stress in different cultivars.

Lipid peroxidation (MDA) was high in both cultivars in the controls. These levels likely reflect germination-associated metabolic activity that promotes the production of reactive oxygen species and represent basal peroxidation in non-stressed seedlings [58,59]. Salt stress increased oxidative damage in both cultivars, with a greater impact in cv. Tetsukabuto, indicating higher sensitivity. Hydropriming (H_2_O) was the most effective attenuator in reducing MDA levels in both cultivars, with a more expressive effect in Tetsukabuto.

In general, increases in MDA and H_2_O_2_^−^ synthesis under salt stress are associated with redox imbalance and damage to cell membranes and protein activity, with severity varying according to stress duration [60]. Considering the high MDA and low H_2_O_2_^−^ levels observed, other reactive oxygen species may also have contributed to membrane peroxidation. For some species, hydropriming has been considered an effective alternative for reducing the effects of abiotic stresses [61].

Alterations in CAT, APX, and SOD indicate that salt stress negatively affected the antioxidant systems of cvs. Tetsukabuto and Adele, albeit with distinct responses. In the control, CAT and APX activities were higher in cv. Adele, whereas SOD was similar between cultivars. In Adele, high CAT and APX activities suggest coordinated ROS detoxification, consistent with the higher protein concentration (83 mg g^−1^ FM protein) and lower peroxide levels, although this was insufficient to avoid lipid peroxidation. In Tetsukabuto, lower protein synthesis (57 mg g^−1^ FM protein) may have contributed to reduced antioxidant efficiency.

Under salinity conditions (SS), CAT and APX activities were reduced in both cultivars, while SOD increased compared with the control, especially in cv. Tetsukabuto. A similar pattern occurred for protein content, with an increase of 132% for Tetsukabuto. These results suggest an antioxidant imbalance, with elevated superoxide not matched by proportional CAT and APX responses. The low peroxide levels under SS may reflect consumption by other pathways or conversion to hydroxyl radicals (OH), consistent with the high MDA concentration, particularly in cv. Tetsukabuto. Such responses are common in plants under salt stress, where osmotic and ionic components induce ROS overproduction, damaging cellular organelles and promoting lipid peroxidation [54].

Among the pre-germinative treatments, in cv. Adele, only SA, AsA, and H_2_O improved CAT activity relative to salt stress (SS), and hydropriming also increased APX activity, possibly due to higher protein levels. In cv. Tetsukabuto, only hydropriming (H_2_O) enhanced both enzymes.

During germination, antioxidant enzymes, such as SOD, CAT, and peroxidase, act in the regulation of free radicals in viable seeds, with catalase being essential for using hydrogen peroxide as a substrate [62]. In the present study, attenuators such as SA, AsA, and H_2_O partially improved CAT activities for Adele, without a proportional reduction in MDA levels, while hydropriming (H_2_O) was efficient for Tetsukabuto. Similar results were observed by Dantas et al. [28], who reported an increase in CAT activity in the embryonic axis of *C. pepo*, cv. Redonda, and *C. maxima*, cv. Coroa, with increased electrical conductivity, while it remained constant in *C. pepo*, cv. Caserta, highlighting cultivar differences.

Hydropriming (H_2_O) for Tetsukabuto and salicylic acid (SA) for Adele were the most efficient at improving CAT activity, enabling the cultivars to cope with stress. Rhaman et al. [53] stated that seed treatment with salicylic acid (SA) helped mitigate abiotic stresses by increasing the activity of antioxidant enzymes, such as catalase.

The reduction in APX is consistent with Dantas et al. [28], who reported decreased APX activity in the embryonic axis under salinity and low activity in pumpkin cotyledons. Similar results were reported in cucurbits, such as watermelon and cucumber, regarding APX reduction [63,64].

The increase in APX in Tetsukabuto under hydropriming suggests an induction of metabolic adjustments that attenuate oxidative damage, while in cv. Adele, salicylic acid contributed to the regulation of this enzyme, favoring H_2_O_2_ detoxification. This result aligns with the findings of Sahu and Sabat [65], who concluded that exogenous application of salicylic acid (SA) modulated intracellular antioxidant enzyme activities, promoting greater plant tolerance to salt stress.

An increase in SOD activity, also observed by Zhu et al. [66], was particularly evident at NaCl levels of 60 mM. The attenuators GA_3_ and AsA were effective in maintaining SOD activity in cultivars Adele and Tetsukabuto, respectively, and were associated with better performance under salt stress. Zhu et al. [66] reported that GA_3_ application attenuated the inhibitory effects of salt stress, promoting increased SOD activity. Finally, Sarker and Oba [67] demonstrated that tolerance to salt stress does not necessarily depend on the joint action of all antioxidant enzymes, with a predominance of enzymes such as SOD and APX and non-enzymatic antioxidants in H_2_O_2_ detoxification in tolerant plants, whereas CAT and GPOX prevail under greater H_2_O_2_ accumulation in sensitive plants. Overall, the results indicate that antioxidant responses are not uniformly integrated across enzymes, suggesting distinct mechanisms protect cells against salt-induced damage.

## 4. Materials and Methods

This research was conducted at the Seed Analysis Laboratory (LAS), part of the Department of Agronomic and Forestry Sciences (DCAF), at the Federal Rural University of the Semi-Arid Region (UFERSA), Mossoró, RN, Brazil (5°11′ S latitude, 37°20′ W longitude, 18 m altitude).

The experiment was conducted in two stages. The first stage aimed to select two pumpkin cultivars, one sensitive and one tolerant to salt stress, and to determine the appropriate salinity level to be used in the second stage. The second stage prioritized the physiological growth responses, osmotic homeostasis, and analysis of the enzymatic defense system of seedlings of two *Cucurbita* spp. cultivars subjected to pre-treatments with attenuators and subsequently exposed to salt stress conditions. The seeds used were purchased from the companies Sakata and Top Seed, operating in the Mossoró region, RN, Brazil.

### 4.1. Stage I

Different *Cucurbita* spp. cultivars were evaluated to represent the genetic diversity of the genus, including five pumpkins (Tetsukabuto, Soberana, Kin, Bahiana Tropical, and Sergipana) and one zucchini (Adele).

Seed water content was determined using the oven method at 105 ± 3 °C for 24 h [68], with two samples of 4.5 ± 0.5 g. Moisture was calculated from the wet mass and expressed as a percentage, with an average of 7.5%.

The experimental design was completely randomized in a 3 × 6 factorial scheme, with four replications of 25 seeds each. The first factor corresponded to three levels of osmotic potential simulating salt stress (0.0, −0.2, and −0.4 MPa), and the second factor consisted of the six pumpkin cultivars.

Seeds were sown on two sheets of paper towel and covered with a third sheet moistened with distilled water (control = 0.0 MPa) and sodium chloride (NaCl) solutions with osmotic potentials of −0.2 and −0.4 MPa [69]. The amount of solution applied was equivalent to twice the dry mass of the paper [68]. The papers were then rolled, placed in plastic bags, and kept in a germination chamber at 25 °C with an 8 h photoperiod.

The variables analyzed were:

(a) First germination count (FGC): performed on the fourth day after sowing; seedlings with developed radicles were counted.

First germination count (FGC%) = (Number of germinated seeds on the 4th day/Total number of seeds) × 100(1)

(b) Germination (G): percentage of seeds germinated on the eighth day after sowing [68].

Germination (G%) = (Number of germinated seeds/Total number of seeds) × 100(2)

(c) Germination speed index (GSI): performed simultaneously with the germination test, counting germinated seeds daily until the eighth day, with the index calculated according to the methodology of Maguire [70].

Germination speed index (GSI) = G_1_/N_1_ + G_2_/N_2_ + … + G_n_/N_n_ (3)

where G_1_, G_2_, …, G_n_ correspond to the number of germinated seeds recorded in the first, second, and last counts, respectively; and N_1_, N_2_, …, N_n_ represent the number of days from sowing to the first, second, and last counts, respectively.

(d) Shoot length (SL) and root length (RL): at the end of the germination test, 10 normal seedlings were randomly selected, and shoot length (measured from the crown to the seedling apex) and root length (measured from the crown base to the root tip) were measured with a ruler graduated in centimeters per seedling.
(e) Shoot dry mass (SDM) and root dry mass (RDM): the seedlings selected for biometric measurements were cut, placed in kraft paper bags, and dried in an oven at 65 °C with forced air circulation. After drying, they were weighed on an analytical balance (0.0001 g), and the results were expressed as g per seedling.

After completion of the germination test, at 8 days after sowing, normal seedlings were collected for the determination of biochemical and physiological variables. Whole seedlings, including the shoot and root, were used for the analyses, with the cotyledons removed prior to sample processing.

For biochemical analyses, pumpkin seedlings were collected and stored in Falcon tubes. Fresh mass was macerated in liquid nitrogen (−196 °C), 0.2 g of the sample was weighed in three replications, and the material was placed in hermetically sealed tubes. Then, 1 mL of 60% alcohol was added, and the tubes were incubated in a water bath at 60 °C for 20 min. After this process, the tubes were centrifuged at 10,000 rpm for 10 min at 4 °C, and the procedure was repeated three times. The supernatant was used to determine the following variables:

(a) Total soluble sugars (TSS): quantified by the anthrone method [71], using a glucose standard curve, with results expressed as mg glucose g^−1^ fresh mass.
(b) Total free amino acids (TFAA): measured by the acid ninhydrin method [72], using a glycine standard curve, with results expressed as μmol glycine/g fresh mass.
(c) Free proline (FP): determined according to the method described by Bates et al. [73], with results expressed as µmol proline/g fresh mass.
(d) Citrulline (CIT): determined by the photometric method, with results expressed as µmol citrulline/mL [74].

### 4.2. Stage II

The effects of seed-applied stress attenuators on tolerant and sensitive *Cucurbita* spp. cultivars under salt stress were assessed. Treatments included hydropriming, gibberellic acid, salicylic acid, and ascorbic acid.

To define the seed hydration period in the stress attenuators, the required soaking time for each cultivar was first determined. Two replicates of 50 seeds were weighed on a digital analytical balance (accuracy 0.001 g) to construct the soaking curve [68]. Seeds were weighed before soaking and at predetermined intervals until primary root emergence. Soaking was performed on germination paper towels using water equal to twice the dry weight of the paper, and seeds were maintained in germination chambers at 25 °C in the dark. The hydration time was set to allow uptake of the salt stress attenuator without reaching primary root protrusion. Accordingly, an 8 h hydroconditioning period was established, corresponding to completion of phase 1 of germination (Figure A1).

The experiment was conducted in a completely randomized design in a 2 × 6 factorial scheme, with four replications of 25 seeds each. The first factor consisted of two cultivars, Adele (tolerant) and Tetsukabuto (sensitive), and the second factor consisted of different combinations of salt stress and pre-treatments with salt stress attenuators, as follows: T1 = 0.0 MPa (control), T2 = −0.4 MPa (salt stress), T3 = −0.4 MPa + hydropriming, T4 = −0.4 MPa + gibberellic acid (50 mg L^−1^), T5 = −0.4 MPa + ascorbic acid (50 mg L^−1^), T6 = −0.4 MPa + salicylic acid (50 mg L^−1^).

The exposure time and concentrations of the salt stress attenuators were defined from the imbibition curve and preliminary tests. Except for hydropriming, all attenuators were used at 50 mg L^−1^ [75]. After preparing the solutions, the seeds were soaked for 8 h, sufficient for substantial water uptake as indicated by the imbibition curve, and then germinated on paper towels moistened with NaCl solutions at −0.4 MPa.

In this stage, in addition to the tests conducted previously, the seedling enzymatic complex was analyzed. After biometric measurements, normal seedlings from each replicate were collected for protein extract preparation.

Protein extraction followed Azevedo et al. [76], with adaptations. Plant material was macerated in liquid nitrogen until it became a fine powder, and 20% PVPP was added. Samples were mixed in a ratio of 1 g of fresh material to 3 mL of 100 mM potassium phosphate buffer (pH 7.5), supplemented with 1 mM EDTA and 3 mM DTT. The extracts were centrifuged at 10,000 rpm for 30 min at 4 °C. The supernatant, kept on ice, was fractionated and stored at −35 °C until enzymatic assays.

The obtained extracts were subjected to the following analyses:

(a) Protein quantification: performed according to the methodology of Bradford [77], using bovine serum albumin (BSA) as a standard. Absorbance was read at 595 nm, and protein content was expressed as mg protein per gram of fresh matter.
(b) Catalase (CAT) activity: determined by the consumption of 35% H_2_O_2_, monitored by spectrophotometry at 240 nm for 1 min [78], with modifications by Azevedo et al. [76]. Enzyme activity was expressed as μmol min^−1^ mg^−1^ protein.
(c) Ascorbate peroxidase (APX) activity: measured according to Nakano and Asada [79], with readings at 290 nm, using an extinction coefficient of 2.8 mM cm^−1^. Results were expressed as μmol of product formed per minute per mg protein.
(d) Superoxide dismutase (SOD) activity: evaluated according to Giannopolitis and Ries [80], using a white light bath. Readings were performed at 560 nm, with results expressed as activity units per mg protein (U SOD mg^−1^ protein).

For hydrogen peroxide quantification and lipid peroxidation, the extract was prepared by macerating 200 mg of fresh matter with 2 mL of 0.1% TCA containing approximately 20% PVPP. After centrifugation, the following analyses were performed:

(a) Hydrogen peroxide (H_2_O_2_): quantified according to Alexieva et al. [81], with potassium iodide and reading in a spectrophotometer at 390 nm, with results expressed as μmol H_2_O_2_ per gram of fresh matter.
(b) Lipid peroxidation: evaluated according to Heath and Packer [82], with malondialdehyde (MDA) production reacting with 2-thiobarbituric acid (TBA). Results were taken at 535 and 600 nm, with results expressed as μmol MDA per gram of fresh matter.

#### Statistical Analysis

Data were subjected to analysis of variance (ANOVA), and means were compared by the Scott–Knott test (*p* ≤ 0.05) to evaluate cultivar and salt stress effects in stage I. This procedure facilitated grouping of the multiple cultivar × salt stress combinations evaluated in the first experimental stage. Subsequently, cluster analysis was performed using Ward’s minimum variance method with Euclidean distance as the dissimilarity measure, using PAST 4 software. For the analysis of salt stress attenuators, data were subjected to ANOVA, and means were compared by Tukey’s test (*p* ≤ 0.05), which controls the family-wise error rate in multiple comparisons. Statistical analyses were performed using SISVAR^®^ software version 5.8 [83].

## 5. Conclusions

Salt stress impaired germination and seedling growth in *Cucurbita* spp., with contrasting cultivar responses.

Adele was more tolerant to salinity, whereas Tetsukabuto was more sensitive and more responsive to seed pre-treatments with stress attenuators.

Hydropriming and gibberellic acid improved specific physiological and growth responses under salt stress.

Stress tolerance was associated with the accumulation of osmolytes such as proline, sugars, and amino acids, as well as with the modulation of the antioxidant system, highlighting the potential of these physiological and biochemical traits as indicators for identifying contrasting responses to salinity and supporting future breeding strategies.

## Figures and Tables

**Figure 1:**
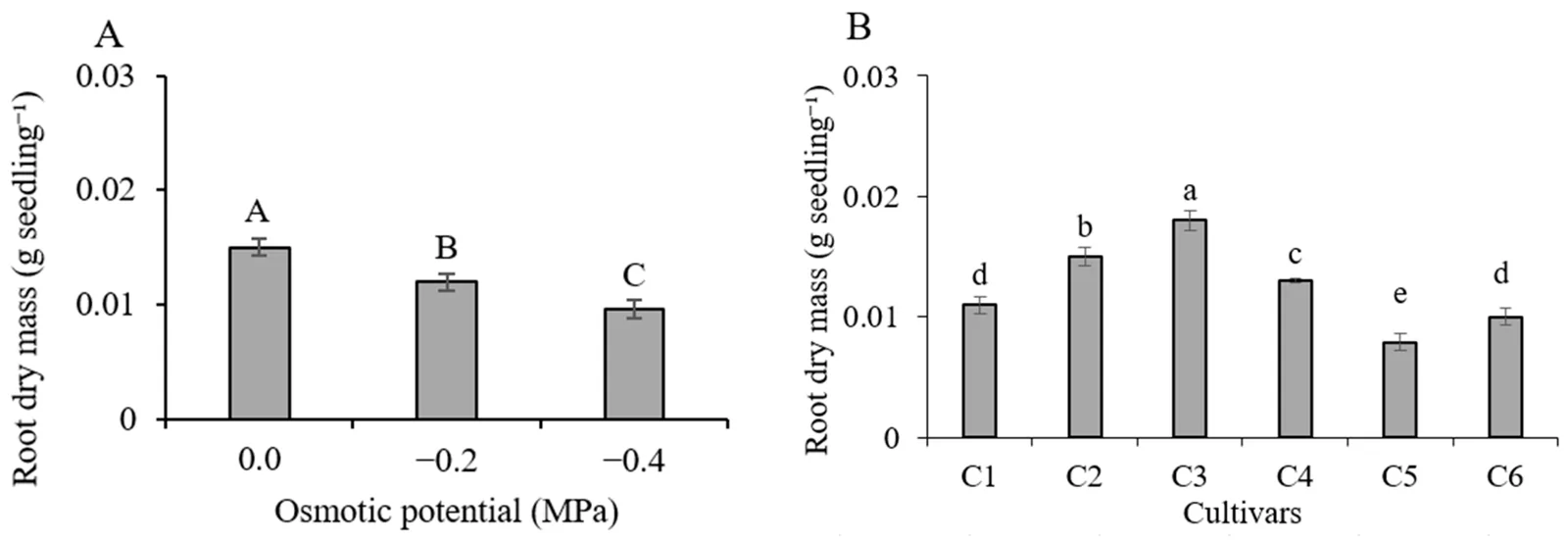
Root dry mass of *Cucurbita* spp. seedlings as a function of different salt stress levels (**A**) and cultivars (**B**). C1 = Tetsukabuto; C2 = Soberana; C3 = Kin; C4 = Adele; C5 = Bahiana Tropical; C6 = Sergipana. Means followed by the same lowercase letter (cultivars) and uppercase letter (osmotic potential) do not differ by the Scott–Knott test at 5% probability.

**Figure 2:**
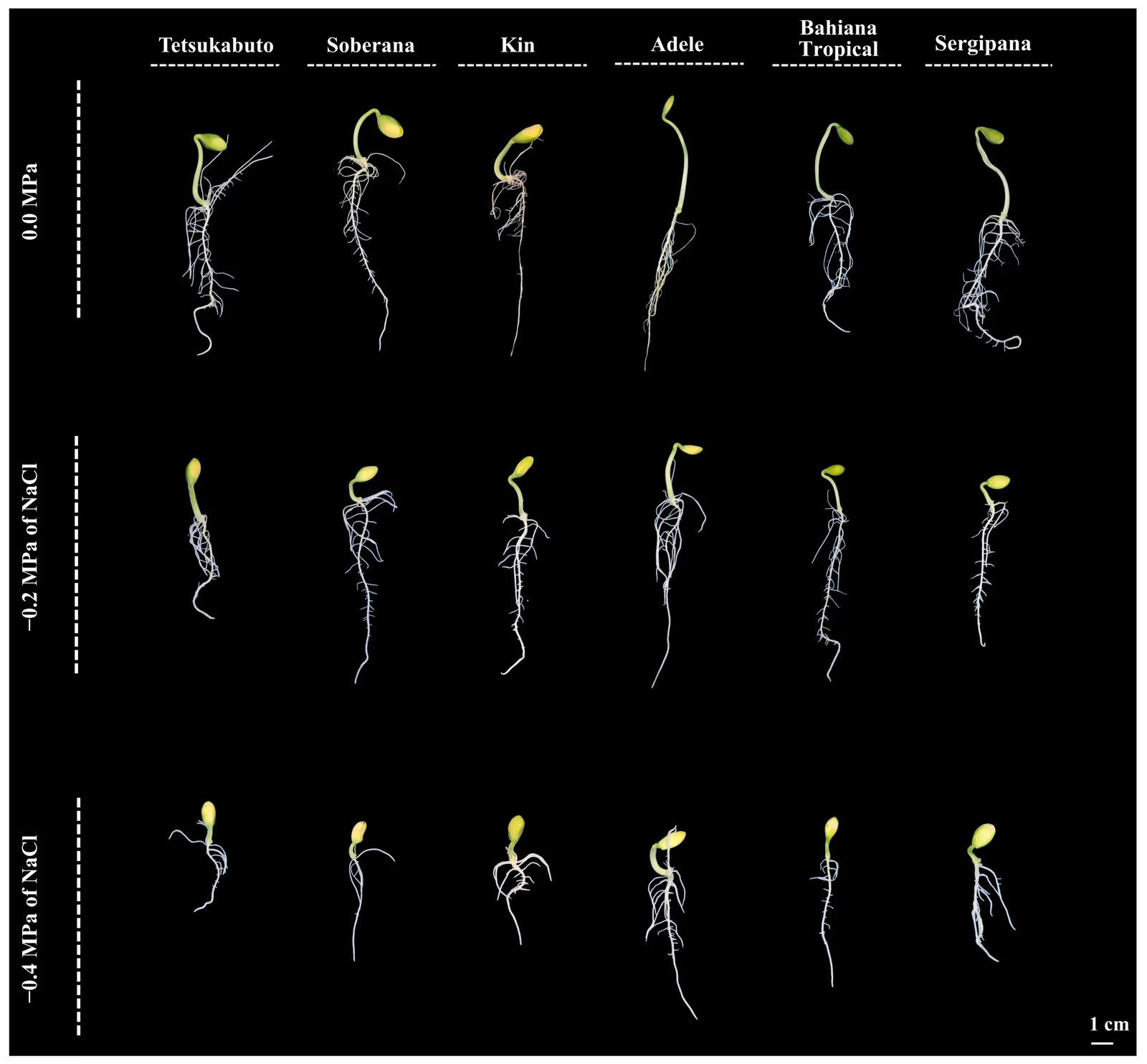
Representative seedlings of six *Cucurbita* spp. cultivars (Tetsukabuto, Soberana, Kin, Adele, Bahiana Tropical, and Sergipana) subjected to three osmotic potentials induced by NaCl (0.0, −0.2, and −0.4 MPa). The images illustrate the differences in shoot and root development among cultivars in response to increasing salt stress. Scale bar = 1 cm.

**Figure 3:**
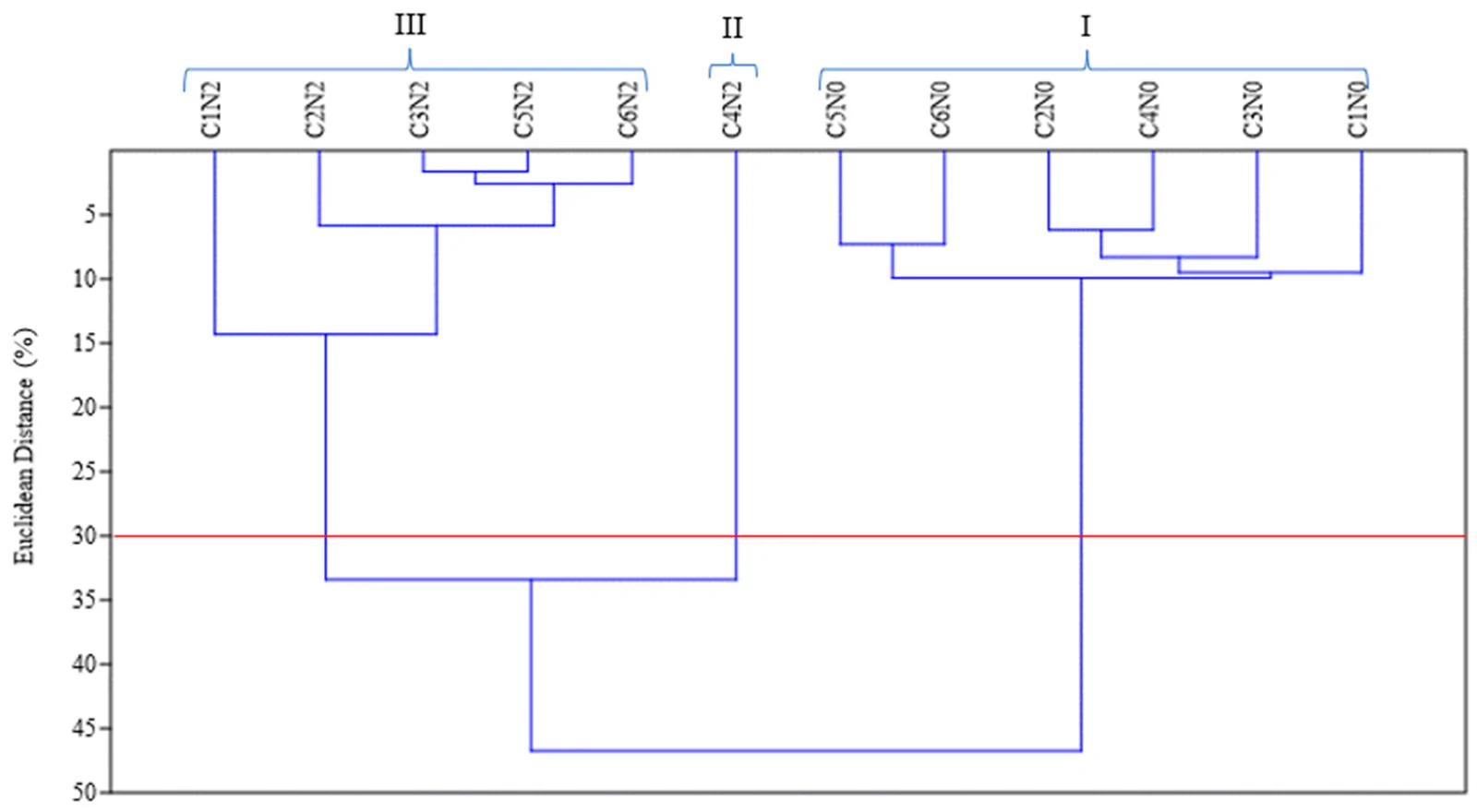
Dissimilarity dendrogram of groups formed by the combination of salt stress levels (N) and pumpkin cultivars (C). N0 = Control; N1 = −0.4 MPa; C1 = Tetsukabuto; C2 = Soberana; C3 = Kin; C4 = Adele; C5 = Bahiana Tropical; C6 = Sergipana. The blue lines represent the hierarchical clustering of treatments based on Euclidean distance, while the red horizontal line indicates the cutoff used to define the groups. Roman numerals I, II, and III identify the three groups formed by the cluster analysis.

**Figure 4:**
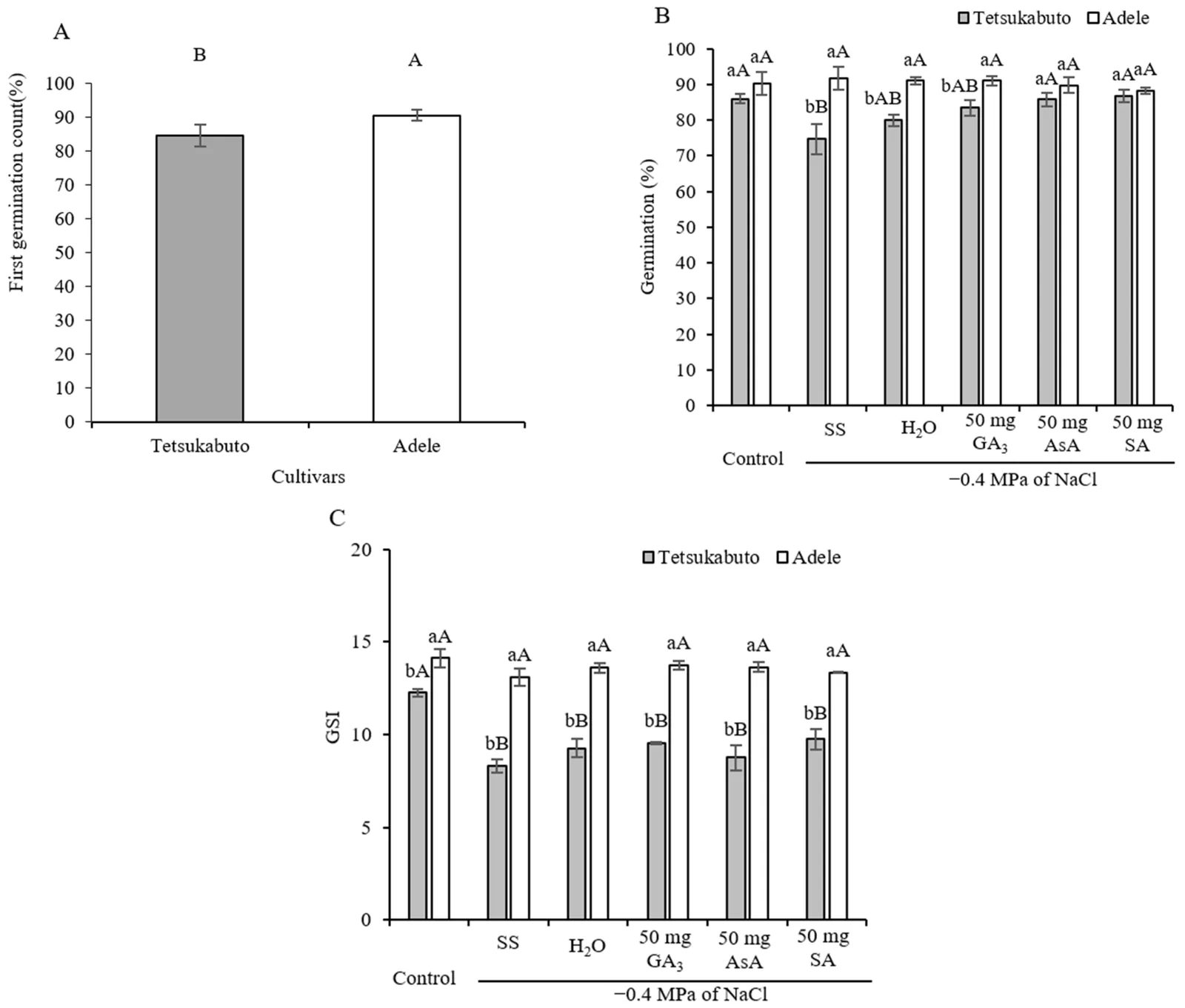
First germination count (**A**), germination (**B**), and germination speed index (**C**) of *Cucurbita* spp. cultivars subjected to pre-germinative treatments and salinity. Control; salinity without attenuators (SS); salinity + hydropriming (H_2_O); salinity + gibberellic acid (GA_3_); salinity + ascorbic acid (AsA); salinity + salicylic acid (SA). Means followed by the same lowercase letter (cultivars) and uppercase letter (pre-germinative treatments) do not differ by Tukey’s test, respectively, at 5% probability.

**Figure 5:**
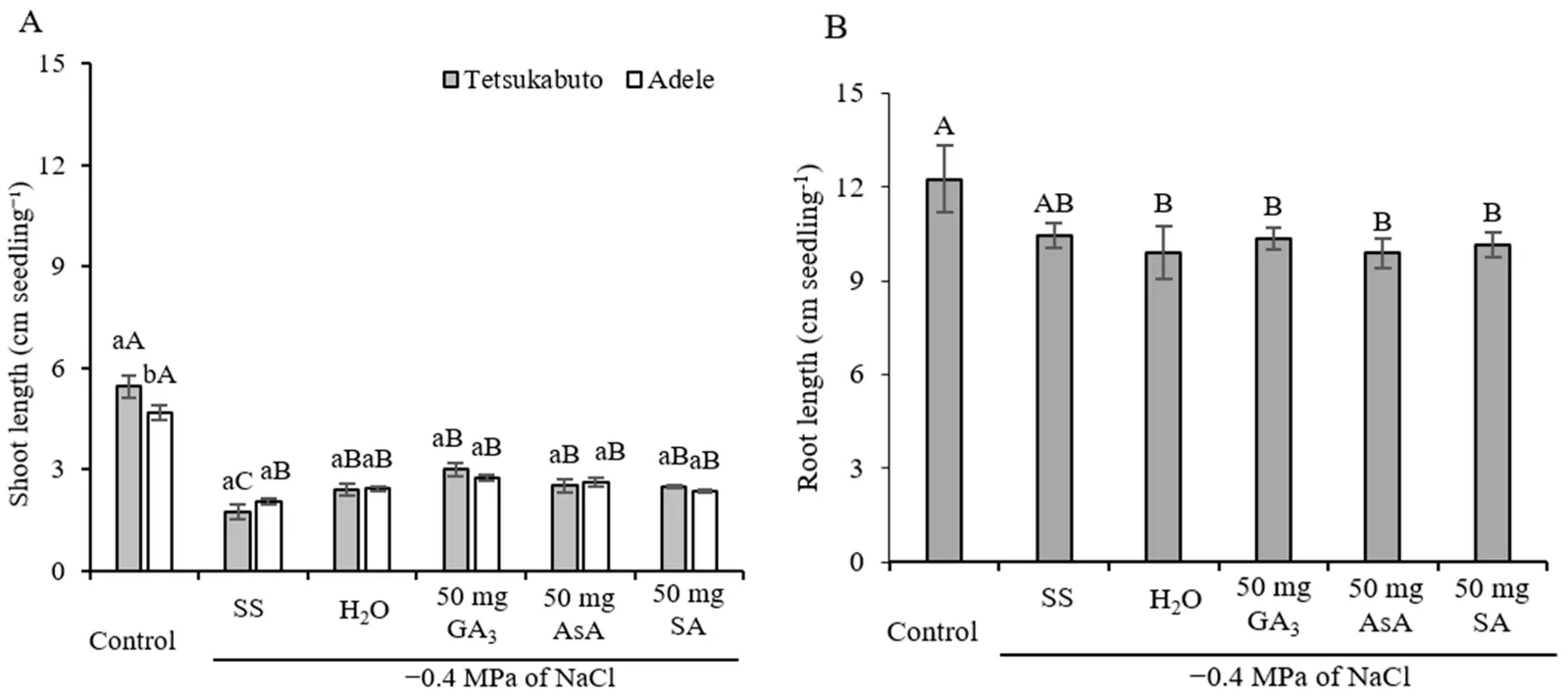
Shoot length (**A**) and root length (**B**) of *Cucurbita* spp. cultivars subjected to pre-germinative treatments and salinity. Control; salinity without attenuators (SS); salinity + hydropriming (H_2_O); salinity + gibberellic acid (GA_3_); salinity + ascorbic acid (AsA); salinity + salicylic acid (SA). Means followed by the same lowercase letter (cultivars) and uppercase letter (pre-germinative treatments) do not differ by Tukey’s test, respectively, at 5% probability.

**Figure 6:**
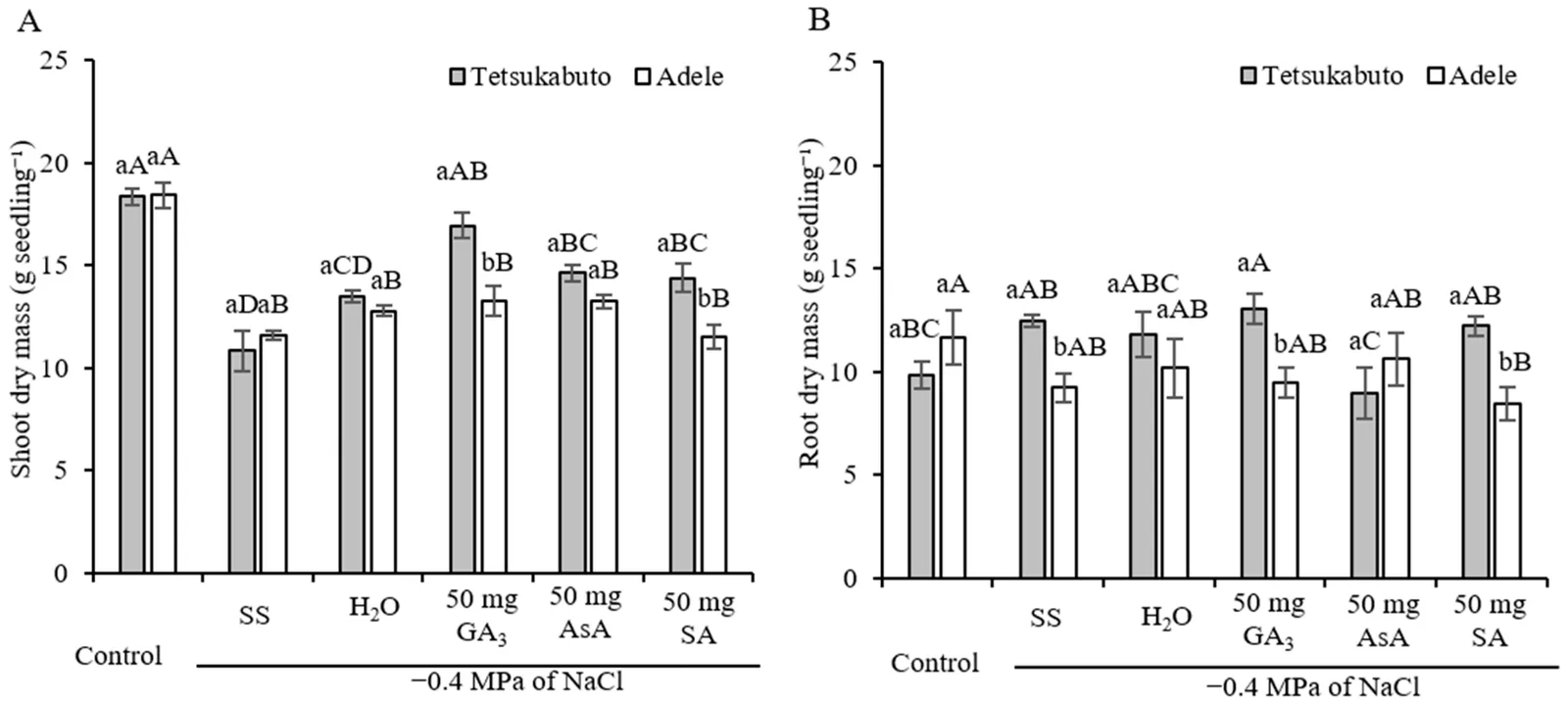
Shoot dry mass (**A**) and root dry mass (**B**) of *Cucurbita* spp. cultivars subjected to pre-germinative treatments and salinity. Control; salinity without attenuators (SS); salinity + hydropriming (H_2_O); salinity + gibberellic acid (GA_3_); salinity + ascorbic acid (AsA); salinity + salicylic acid (SA). Means followed by the same lowercase letter (cultivars) and uppercase letter (pre-germinative treatments) do not differ by Tukey’s test, respectively, at 5% probability.

**Figure 7:**
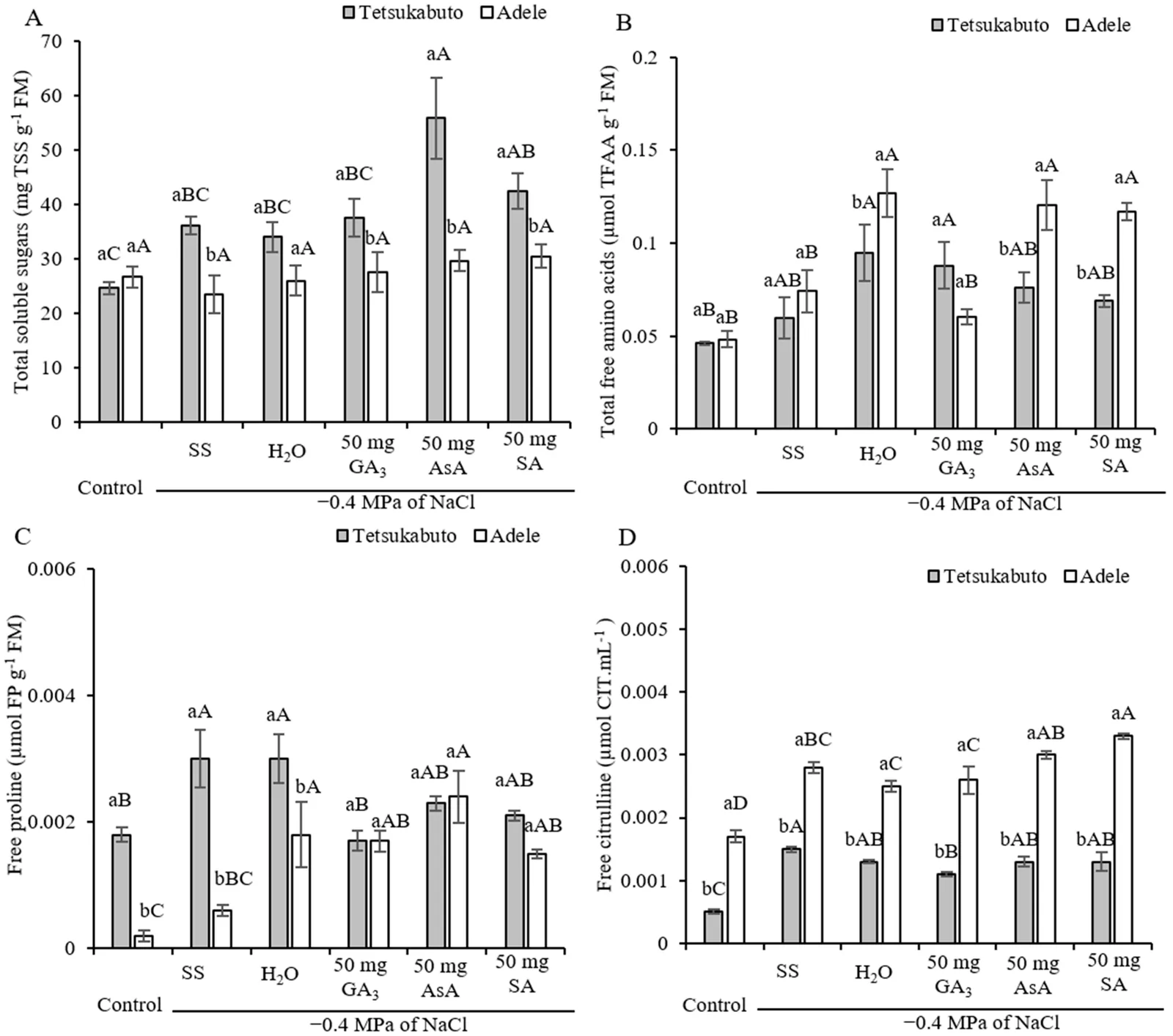
Total soluble sugars (**A**), free amino acids (**B**), free proline (**C**), and citrulline (**D**) of *Cucurbita* spp. cultivars subjected to pre-germinative treatments and salinity. Control; salinity without attenuators (SS); salinity + hydropriming (H_2_O); salinity + gibberellic acid (GA_3_); salinity + ascorbic acid (AsA); salinity + salicylic acid (SA). Means followed by the same lowercase letter (cultivars) and uppercase letter (pre-germinative treatments) do not differ by Tukey’s test, respectively, at 5% probability.

**Figure 8:**
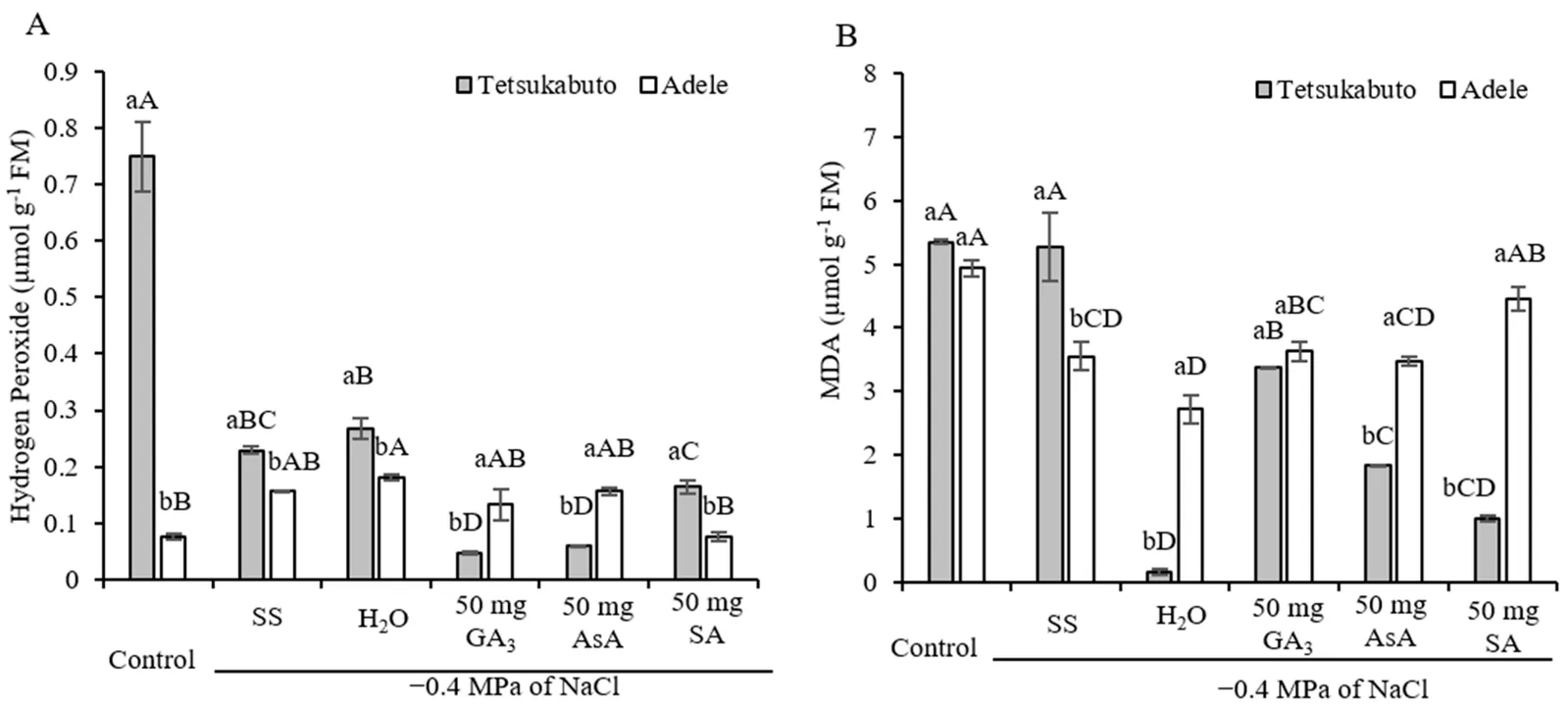
Lipid peroxidation expressed by hydrogen peroxide (**A**) and malondialdehyde (**B**) content of *Cucurbita* spp. cultivars subjected to pre-germinative treatments and salinity. Control; salinity without attenuators (SS); salinity + hydropriming (H_2_O); salinity + gibberellic acid (GA_3_); salinity + ascorbic acid (AsA); salinity + salicylic acid (SA). Means followed by the same lowercase letter (cultivars) and uppercase letter (pre-germinative treatments) do not differ by Tukey’s test, respectively, at 5% probability.

**Figure 9:**
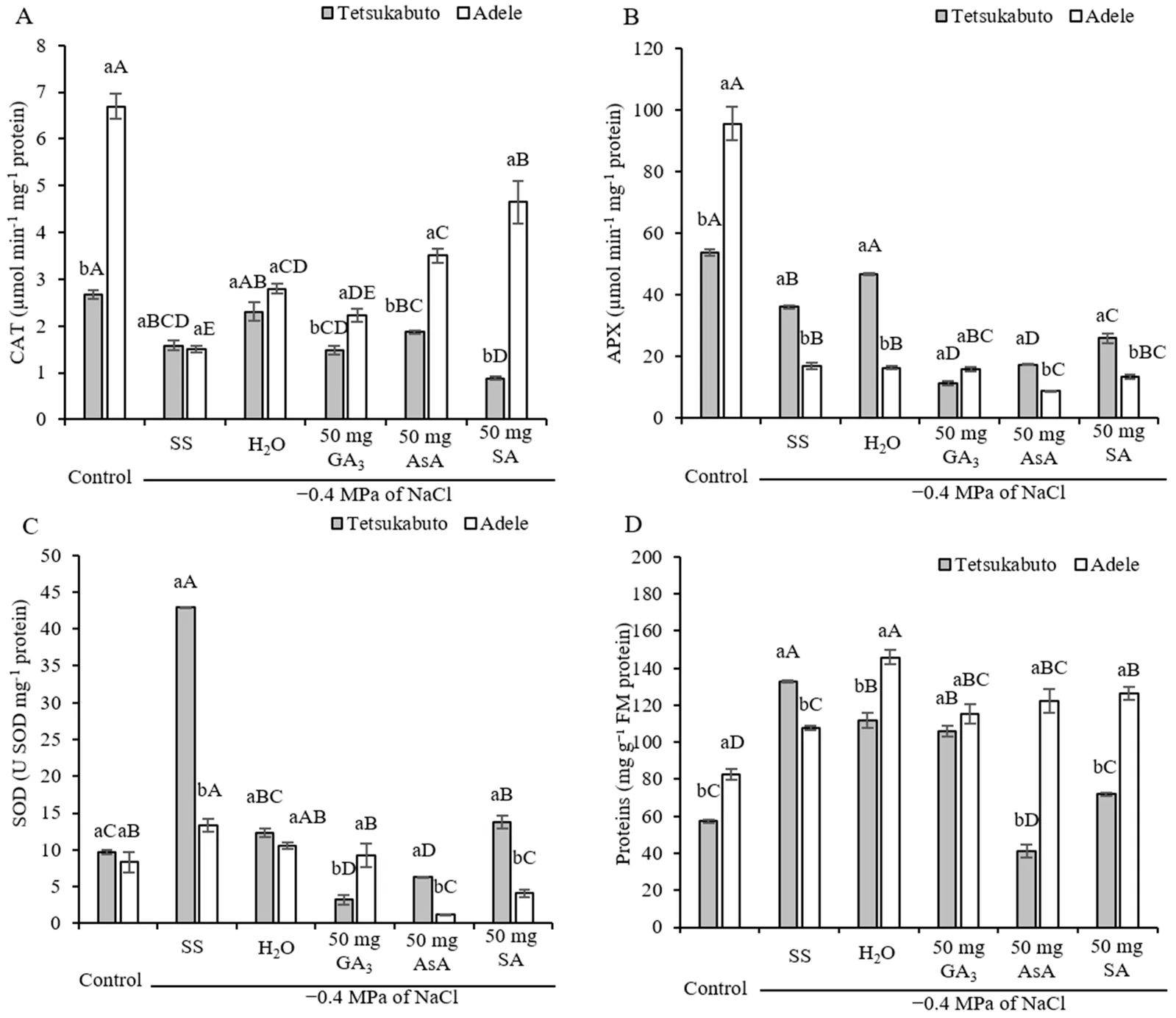
Activity of the enzymes catalase—CAT (**A**), ascorbate peroxidase—APX (**B**), superoxide dismutase—SOD (**C**), and proteins (**D**) of *Cucurbita* spp. cultivars subjected to pre-germinative treatments and salinity. Control; salinity without attenuators (SS); salinity + hydropriming (H_2_O); salinity + gibberellic acid (GA_3_); salinity + ascorbic acid (AsA); salinity + salicylic acid (SA). Means followed by the same lowercase letter (cultivars) and uppercase letter (pre-germinative treatments) do not differ by Tukey’s test, respectively, at 5% probability.

**Figure 10:**
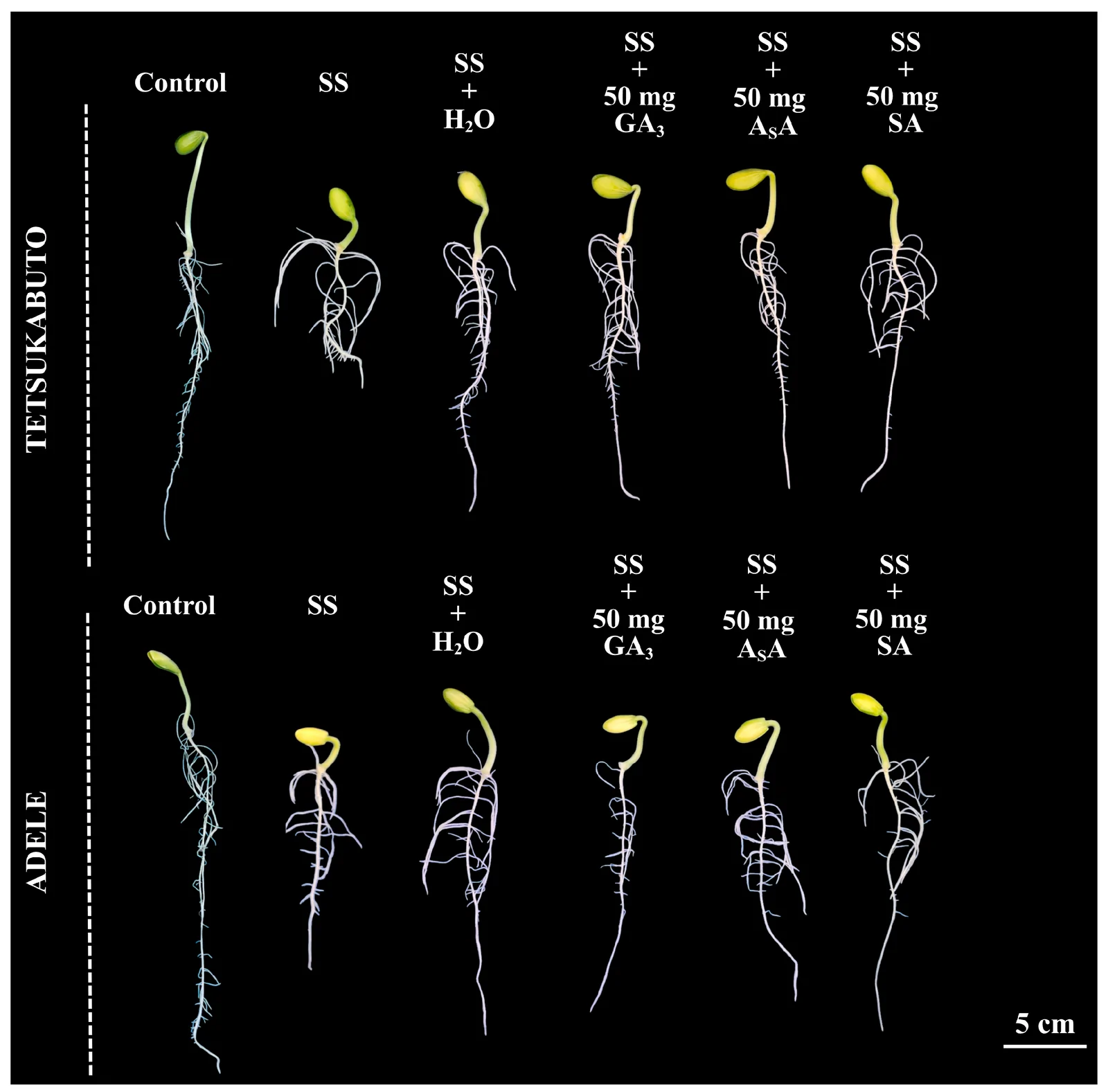
Representative visual aspect of *Cucurbita* spp. cultivar seedlings subjected to pre-germinative treatments and salinity. Control; salinity without attenuators (SS); salinity + hydropriming (H_2_O); salinity + gibberellic acid (GA_3_); salinity + ascorbic acid (AsA); salinity + salicylic acid (SA). Images illustrate morphological differences and development among treatments. Scale bar: 5 cm.

**Table 1:** Summary of analysis of variance for first germination count (FGC), germination (G), germination speed index (GSI), shoot length (SL), root length (RL), shoot dry mass (SDM), and root dry mass (RDM) (g seedling^−1^) of *Cucurbita* spp. cultivars as a function of salt stress.

Sources of Variation	Mean Square						
	FGC	G	GSI	SL	RL	SDM	RDM
	(%)	(%)	-	(cm Seedling^−1^)		(g Seedling^−1^)	
Cultivars (Cult.)	4092.22 **	740.62 **	34.90 **	4.66 **	5.35 **	0.000127 **	0.000179 **
Salt stress (SS)	46,322.88 **	76.22 ns	33.92 **	91.69 **	71.85 **	0.000456 **	0.000239 **
Cult. × SS.	1331.95 **	148.75 **	2.17 **	1.24 **	4.57 **	0.000023 **	0.000005 ns
CV (%)	13.04	5.55	4.99	18.85	10.57	16.89	15.32

ns: non-significant. **: significant at 1% probability by the F test. CV: coefficient of variation.

**Table 2:** Means of first germination count (FGC), germination (G), and germination speed index (GSI) of *Cucurbita* spp. cultivars as a function of salt stress.

Cultivars	FGC (%)			G (%)			GSI		
	0.0 MPa	−0.2 MPa	−0.4 MPa	0.0 MPa	−0.2 MPa	−0.4 MPa	0.0 MPa	−0.2 MPa	−0.4 MPa
Tetsukabuto	81 bA	0 bB	0 bB	86 bA	61 bC	76 bB	8.88 cA	7.06 cB	6.30 cC
Soberana	93 aA	0 bB	0 bB	99 aA	93 aB	88 aB	10.13 bA	7.46 bB	6.56 cC
Kin	87 aA	0 bB	0 bB	93 aA	96 aA	93 aA	10.55 bA	7.67 bB	6.52 cC
Adele	93 aA	94 aA	33 aB	94 aA	98 aA	95 aA	11.94 aA	12.11 aA	11.45 aA
Bahiana Tropical	79 bA	0 bB	0 bB	92 aA	92 aA	92 aA	8.48 cA	7.46 bB	7.28 bB
Sergipana	84 bA	0 bB	0 bB	87 bA	90 aA	93 aA	8.54 cA	6.81 cB	6.58 cB

Means followed by the same lowercase letter in the column and the same uppercase letter in the row, for the same indicator, do not differ according to the Scott–Knott test at the 5% probability level.

**Table 3:** Means of shoot length (SL), root length (RL), and shoot dry mass (SDM) (g seedling^−1^) of *Cucurbita* spp. cultivars as a function of salt stress.

Cultivars	SL (cm Seedling^−1^)			RL (cm Seedling^−1^)			SDM (g Seedling^−1^)		
	0.0 MPa	−0.2 MPa	−0.4 MPa	0.0 MPa	−0.2 MPa	−0.4 MPa	0.0 MPa	−0.2 MPa	−0.4 MPa
Tetsukabuto	4.82 bA	1.86 cB	1.12 aB	11.03 aA	9.03 cB	7.91 aB	0.017 aA	0.0091 dB	0.006 bB
Soberana	5.77 aA	2.10 cB	1.23 aC	11.51 aA	10.51 cA	8.89 aB	0.020 aA	0.011 cB	0.008 aC
Kin	3.60 cA	2.75 bB	1.12 aC	10.10 aB	12.06 bA	8.58 aB	0.018 aA	0.015 bA	0.008 aB
Adele	6.45 aA	4.24 aB	1.96 aC	11.56 aA	11.50 bA	8.30 aB	0.017 aB	0.020 aA	0.010 aC
Bahiana Tropical	5.68 aA	3.14 bB	1.24 aC	12.64 aA	13.82 aA	7.59 aB	0.010 bA	0.008 dA	0.004 bB
Sergipana	4.97 bA	2.80 bB	1.39 aC	10.96 aA	10.43 cA	8.34 aB	0.011 bA	0.009 dA	0.005 bB

Means followed by the same lowercase letter in the column and the same uppercase letter in the row, for the same indicator, do not differ according to the Scott–Knott test at the 5% probability level.

**Table 4:** Summary of analysis of variance for total soluble sugars (TSS), total free amino acids (TFAA), free proline (FP), and free citrulline (CIT) of *Cucurbita* spp. cultivars as a function of salt stress.

Sources of Variation	Mean Square			
	TSS	TFAA	FP	CIT
	(mg g^−1^ FM)	(μmol g^−1^ FM)		(μmol mL^−1^)
Cultivars (Cult.)	150.9551 **	0.0017 **	0.000032 **	0.000001 **
Salt stress (SS)	190.5302 **	0.0058 **	0.000051 **	0.000003 **
Cult. × SS.	51.4433 **	0.0040 **	0.000004 **	0.00000 **
CV (%)	13.41	18.06	16.62	11.62

**: significant at 1% probability by the F test. CV: coefficient of variation.

**Table 5:** Means of total soluble sugars (TSS) and total free amino acids (TFAA) in seedlings of *Cucurbita* spp. cultivars as a function of salt stress.

Cultivars	TSS (mg g^−1^ FM)			TFAA (μmol g^−1^ FM)		
	0.0 MPa	−0.2 MPa	−0.4 MPa	0.0 MPa	−0.2 Mpa	−0.4 MPa
Tetsukabuto	11.87 aB	20.96 aA	3.7888 cC	0.126 aA	0.066 aB	0.063 cB
Soberana	9.96 aB	14.74 cA	11.5249 aB	0.057 cB	0.085 aA	0.082 cA
Kin	11.28 aB	16.54 bA	7.2275 bC	0.090 bA	0.082 aA	0.099 bA
Adele	6.86 bA	7.27 dA	6.9832 bA	0.049 cC	0.086 aB	0.108 bA
Bahiana Tropical	2.65 cB	7.34 dA	7.5431 bA	0.031 dC	0.057 bB	0.145 aA
Sergipana	2.47 cB	5.71 dA	4.6609 cA	0.058 cA	0.032 cB	0.076 cA

Means followed by the same lowercase letter in the column and the same uppercase letter in the row, for the same indicator, do not differ according to the Scott–Knott test at the 5% probability level.

**Table 6:** Means of free proline (FP) and citrulline (CIT) in seedlings of *Cucurbita* spp. cultivars as a function of salt stress.

Cultivars	FP (μmol g^−1^ FM)			CIT (μmol mL^−1^)		
	0.0 MPa	−0.2 MPa	−0.4 MPa	0.0 MPa	−0.2 Mpa	−0.4 MPa
Tetsukabuto	0.0059 aB	0.0049 aC	0.0072 aA	0.0018 bA	0.0018 bA	0.0021 bA
Soberana	0.0026 cC	0.0041 bB	0.0063 bA	0.0016 bB	0.0021 bA	0.0022 bA
Kin	0.0041 bB	0.0035 bB	0.0058 bA	0.0018 bB	0.0027 aA	0.0023 bA
Adele	0.0010 dB	0.0007 dB	0.0029 cA	0.0019 bC	0.0027 aB	0.0036 aA
Bahiana Tropical	0.0017 dC	0.0039 bB	0.0078 aA	0.0020 bB	0.0024 aB	0.0034 aA
Sergipana	0.0014 dB	0.0025 cA	0.0031 cA	0.0026 aA	0.0021 bA	0.0023 bA

Means followed by the same lowercase letter in the column and the same uppercase letter in the row, for the same indicator, do not differ according to the Scott–Knott test at the 5% probability level.

**Table 7:** Summary of analysis of variance for first germination count (FGC), germination (G), germination speed index (GSI), shoot length (SL), root length (RL), shoot dry mass (SDM), and root dry mass (RDM) (g seedling^−1^) of *Cucurbita* spp. cultivars as a function of attenuator treatments.

Sources of Variation	Mean Square						
	FGC	G	GSI	SL	RL	SDM	RDM
	(%)	(%)	-	(cm Seedling^−1^)		(g Seedling^−1^)	
Cultivars (Cult.)	432.00 **	682.520833 **	192.400208 **	0.187500 ns	4.025208 ns	20.150208 **	25.070752 **
Attenuator treatments (AT)	9.433333 ns	27.687500 ns	5.589208 **	9.987500 **	6.360875 **	47.865208 **	2.184842 ns
Cult. × AT	45.00 ns	64.520833 *	2.397208 **	0.277000 *	1.134208 ns	5.758208 *	13.390612 **
CV (%)	6.26	5.39	6.2	11.82	11.39	10.65	13.72

ns: non-significant. ** and *: significant at 1 and 5% probability by the F test, respectively. CV: coefficient of variation.

**Table 8:** Summary of analysis of variance for total soluble sugars (TSS), total free amino acids (TFAA), free proline (FP), and free citrulline (CIT) of *Cucurbita* spp. cultivars as a function of attenuator treatments.

Sources of Variation	Mean Square			
	TSS	TFAA	FP	CIT
	(mg g^−1^ FM)	(μmol g^−1^ FM)		(μmol mL^−1^)
Cultivars (Cult.)	1494.572 **	0.004276 **	0.000011 **	0.000026 **
Attenuator treatments (AT)	289.154 **	0.004342 **	0.000002 **	0.000002 **
Cult. × AT	165.788 **	0.00165 **	0.000002 **	1.92945 × 10^−7^ **
CV (%)	20.4	23.62	28.82	9.77

**: significant at 1% probability by the F test. CV: coefficient of variation.

**Table 9:** Summary of analysis of variance with mean squares for the variables: proteins, catalase (CAT), ascorbate peroxidase (APX), superoxide dismutase (SOD), hydrogen peroxide (HP), and malondialdehyde (MDA) of *Cucurbita* spp. cultivars as a function of attenuator treatments.

Sources of Variation	Mean Square					
	PROTEIN (mg g^−1^ FM Protein)	CAT	APX	SOD	MDA	HP
		µmol min^−1^ mg^−1^ de Protein		U SOD mg^−1^ de Protein	µmol g^−1^ FM	
Cultivars (Cult.)	10,702.8395 **	37.3527 **	199.2186 **	567.6296 **	11.2162 **	0.1823 **
Attenuator treatments (AT)	4125.3601 **	9.6374 **	4255.7247 **	603.2542 **	14.2334 **	0.1164 **
Cult. × AT	2689.4385 **	6.0716 **	1272.8309 **	302.1287 **	7.5461 **	0.1603 **
CV (%)	6.84	13.63	11.71	13.93	11.96	21.95

**: significant at 1% probability by the F test. CV: coefficient of variation.

**Figure A1:**
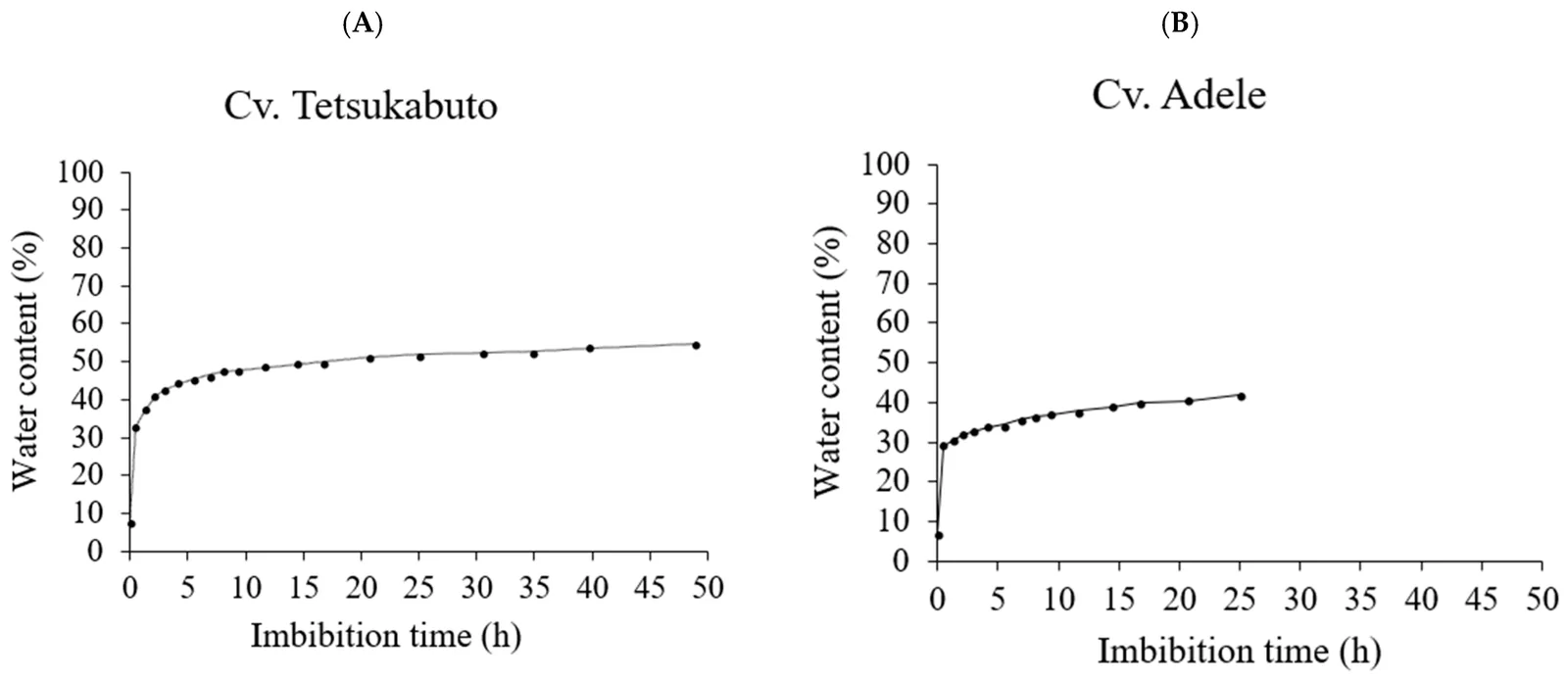
Determination of the imbibition curve of *Cucurbita* spp. cultivar seeds at 25 °C: (**A**) Tetsukabuto; (**B**) Adele.

## Data Availability

Data is contained within the article.
